# Alterations of Mast Cells in the Esophageal Mucosa of the Patients With Non-Erosive Reflux Disease

**DOI:** 10.4021/gr284w

**Published:** 2011-03-20

**Authors:** Yue Yu, Xiping Ding, Qiaomin Wang, Li Xie, Wen Hu, Ke Chen

**Affiliations:** a Division of Gastroenterology, Affiliated Provincial Hospital, Anhui Medical University, Hefei 230001, China; b Department of Pathology, Affiliated Provincial Hospital, Anhui Medical University, Hefei 230001, China

**Keywords:** Non-erosive reflux disease, Esophagus, Mast cell

## Abstract

**Background:**

Mast cells (MCs) are widely distributed in the gastrointestinal tract, which could be involved in visceral hypersensitivity and gut dysmotility. Whether esophageal MCs play a role in non-erosive reflux disease (NERD) has yet to be determined. The aim of this study was to characterize esophageal MCs distribution, degranulation, and ultrastructure.

**Methods:**

The esophageal mucosa at 5 cm above the end of esophagus was obtained from 26 NERD and 14 healthy volunteers (control) by gastroscopy. Immunohistochemistry was performed and average MC counts per high-power field (HPF) and the percentage of degranulated MCs were obtained. The ultrastructure of MCs was observed by transmission electron microscope (TEM).

**Results:**

More MCs were observed in NERD (7.23 ± 2.41 cells/HPF) as compared with controls (3.79 ± 1.67 cells/HPF) (P < 0.01) and the percentage of degranulated MCs in NERD was also significantly higher than controls (26.85 ± 8.79% vs 11.5 ± 4.18%, P < 0.01). Under TEM, more Golgi apparatus, mitochondria and endoplasmic reticulum were found in MCs in patients with NERD. Special secreting particles were also found in cytoplasm, more vacuoles were left after MCs degranulation in patients with NERD.

**Conclusions:**

Our results indicate that increased numbers of MCs and MCs activation may be involved in the pathogenesis of NERD.

## Introduction

Gastroesophageal reflux disease (GERD) is a highly prevalent (10% - 20% of population) condition that may have a significant impact on quality of life. The etiopathogenesis of this disease is multifactorial, and the main factor responsible for it is a dysfunction of the lower esophageal sphincter [1, 2]. Most patients with GERD do not have erosive esophagitis (EE), and this situation is known as GERD with negative endoscopy or non-erosive reflux disease (NERD). NERD was previously considered a mild/early type of EE that would progress to severe EE. However, nowdays this condition is not considered a mild or initial presentation of GERD, but as a form of GERD in its own right [3]. In addition, NERD is significantly more refractory to treatment than EE [4]. Therefore, NERD is a more heterogeneous condition from a pathogenic perspective than GERD with esophagitis.

Mast cells (MCs) are widely distributed in the gastrointestinal tract, which have been implicated in various conditions where gastrointestinal motility is altered [5]. MCs produce histamine and serotonin, both of which can stimulate enteric smooth muscle contraction [6]. In other conditions MCs are associated with impaired motility, often due to deleterious effects of mast cell proteases on the interstitial cells of Cajal [7] or on the enteric nervous system [5].

On the other hand, MCs could be responsible for the altered visceral perception found in patients with IBS. Mucosal MCs in the terminal ileum [8], colon [9] and rectum [10] have been proposed to be significantly elevated in most patients with IBS and the close proximity of the mucosal MCs to the enteric nerves [11] suggests their potential involvement in the induced changes in nerve function and the development of visceral hypersensitivity [9].

Consequently, on the basis of these observations, the aim of the present study was firstly to assess the esophageal mucosal MCs infiltration and activation in NERD and secondly, to characterize the esophageal mucosal MCs under the transmission electron microscope.

## Materials and Methods

### Subjects

From October 2008 to February 2009, we studied 26 patients with NERD and endoscopically confirmed normal-appearing esophageal mucosa who visited the outpatient Department of gastroenterology in affiliated Provincial Hospital, Anhui medical university. They included 10 men and 16 women, aged between 21 and 53 years (mean, 32.6 years). The diagnosis of GERD was made by the Chinese version of the Reflux Diagnostic Questionnaire (RDQ) [12]. A subject with GERD symptoms was defined according to the RDQ score (≥ 12), and then was examined by upper gastrointestinal endoscopy. Patients who were suspicious of having GERD but without evidence of reflux esophagitis (RE) were diagnosed as having NERD. None of these patients had been treated with nonsteroidal anti-inflammatory drugs, proton pump inhibitors, histamine H_2_-receptor antagonists, anti-cholinergic agents or antibiotics within 4 weeks prior to the present study. Furthermore, patients with severe concomitant diseases, prior esophageal or gastric surgery, liver cirrhosis, malignant tumor (e.g. gastric cancer), peptic ulcer diseases and comorbid conditions that might interfere with esophageal or gastric motility including diabetes mellitus, systemic sclerosis and neurological disorders were excluded. As a control group, we recruited 14 asymptomatic subjects (8 females, 6 males aged 20 - 55 years, mean 34.2 years) with no hiatal hernia or any lesions in the esophagus, stomach and duodenum at endoscopy for a health check-up.

All subjects underwent gastroscopy, and 3 biopsies were taken from the esophageal mucosa at 5 cm above the end of esophagus in each case, 2 biopsies for routine hematoxylin and eosin (HE) histology and immunohistochemistry, 1 biopsy for transmission electron microscope (TEM, JEOL-1200EX Biosystem, Tokyo, Japan). This study was approved by the Review Board and Ethics Committee of affliated Provincial Hospital, Anhui medical university.

### Questionnaire

The gastroesophageal reflux questionnaire, a self-report instrument that to evaluate reflux-associated symptoms during the prior month, included the Chinese version of the Reflux Diagnostic Questionnaire (RDQ) [12] and items concerning the demographic characteristics and probable risk factors for GERD. It comprised the following parts: (1) general information: gender and age, (2) the Chinese version of the Reflux Diagnostic Questionnaire (RDQ) whose framework was based on a validated questionnaire previously published. The symptoms suggestive of GERD in the RDQ included heartburn, substernal chest pain, acid and food regurgitation. The following definitions were used to identify the symptoms in the RDQ: (1) heartburn, a burning sensation located beneath the sternum; (2) substernal chest pain: any pain felt inside in the chest but not including heartburn or any pain that is primarily originated from the abdomen; (3) acid regurgitation, a bitter- or sour-tasting fluid coming into the throat or mouth; and (4) food regurgitation, unpleasant movement of material upwards from the stomach but not vomit. Each symptom was scored according to the frequency and severity (5-point scale). The highest score for one subject was 40. The frequency was measured according to the following scale: 0, no symptom in the past month; 1, less than once a week; 2, once a week; 3, two to three days a week; 4, four to five days a week; and 5, almost daily. Symptom severity was assessed on the following scale: 0, none; 1, very mild (symptoms can be easily ignored unless reminded of them); 2, mild (between 1 and 3); 3, moderate (symptoms are obvious and sufficient to influence normal activities, and occasionally need treatment); 4, severe (between 3 and 5); and 5, very severe (symptoms are obvious and sufficient to influence normal activities, and need long-term medication).

### Stainning for MCs

Esophageal mucosal MCs were stained using a monoclonal antibody against the human mast cell protease tryptase. Biopsy specimens were fixed in 10% neutral buffered formalin and processed for either HE histology or immunohistochemistry.

For the latter, paraffin-embedded specimens were cut with a microtome at 6 µm thickness, and de-waxed and re-hydrated before treated with 0.5% H_2_O_2_ to quench the endogenous peroxidase. Antigen retrieval was performed on paraffin sections prior to immunostaining by heating the slides in citrate buffer (pH 6.0). Both whole mount preparations and paraffin sections were then washed for 30 min in phosphate buffered saline (PBS; 0.05 mol/L, pH 7.4, with 0.3% Triton X-100). Non-specific antibody binding was reduced by incubating the tissues in 1% bovine serum albumin for 1 h at room temperature before addition of the primary antibodies. The slides were then incubated in monoclonal anti-human mast cell protease tryptase (1 : 200; Santa Cruz, CA, USA) for 18 h at 4 °C, washed in PBS for10 min and then incubated for 30 min with biotinylated anti-rabbit IgG (Vector Laboratories, Burlingame, CA, USA). After the slides had been washed in PBS for 10 min, they were incubated with streptavidin-biotin complex conjugated with horseradish peroxidase (Santa Cruz, CA, USA) for 30 min. The slides were developed in diammobenzidine-hydrogen peroxide substrate (Sigma, St. Louis, MO, USA) for 10 min rinsed in PBS and washed in tap water for 5 min. Sections were then counterstained with hematoxylin, dehydrated, cleared and then mounted in DPX mountant (BDH).

Control sections were treated with rabbit non-immune serum instead of the originally used primary antibody. These acted as control sections. Controls for the specificity of antisera consisted of incubation of the tissue with normal rabbit serum substituted for the primary antiserum.

### Quantification of MCs and MCs degranulation

All histological sections were evaluated by an expert pathologist under a light microscope. Pictures were taken through a digital camera (Sony 3CCD, Model no. DXC-930; Tokyo, Japan) attached to the microscope. Immunohistochemically positive-stained MCs were counted in five consecutive non-overlapping fields of view with area of 0.024 mm^2^ at × 400 magnification. Individual numbers represent immunohistochemically positive MCs per unit area (0.024 mm^2^). Counting was performed by the pathologist blinded to the histological section’s group of origin, with each number expressed with “mean ± SE”. MCs degranulation was determined as a loss of MC membrane integrity with extrusion of intracellular granules to the extracellular space or MCs completely lacking in intracellular granules as described previously [13].

### Electron microscopy

Biopsy specimens were placed in ice-cold physiological solution. Tissues were cut and fixed by immersion in 2.5% glutaraldehyde in 0.075 mol/L sodium cacodylate buffer (pH 7.4, containing 4.5% sucrose and 1 mmol/L CaCl_2_) for 6 h at room temperature. After primary fixation, 1 cm wide pieces were cut and immersed in the same fixative for an additional fixation overnight at 4 °C, then, all tissues were washed in 0.1 mol/L cacodylate buffer, containing 6% sucrose and 1.24 mmol/L CaCl_2_ (pH 7.4) at 4 °C. They were postfixed with 1% osmium tetroxide (OsO_4_) in 0.05 mol/L cacodylate buffer (pH 7.4) for 90 min, stained with saturated uranyl acetate for 60 min, dehydrated in graded ethanol and propylene oxide, and embedded in Epon-Araldite (Marivac Ltd, Halifax, NS, Canada). Following this, ultra thin sections were cut, mounted on 100 mesh grids, and double stained with uranyl acetate and lead citrate. The grids were observed with a transmission electron microscope at 80 kV.

### Statistical analysis

Data are expressed as the mean ± SE. One-way analysis of variance (ANOVA) was conducted with the SPSS 13.0 software to compare the number of mast cells between the two groups. P < 0.05 was considered statistically significant.

## Results

### Esophageal mucosal MCs distribution and morphology

**Figure 1:**
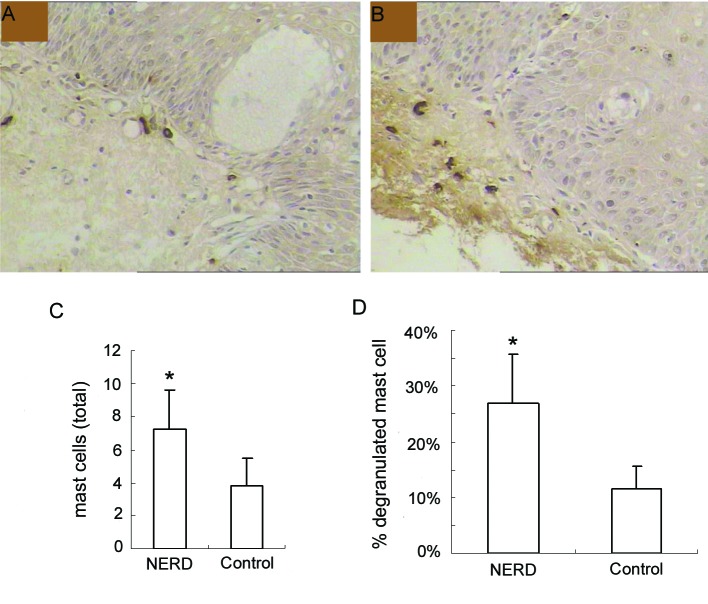
Immunohistochemical analysis of mast cells at the esophageal mucosa related to ‘NERD’ and ‘control’. (A), control, and (B), NERD patients, illustrate the immunohistochemical staining of mast cells (tryptase immunostain, brown cells). Slides shown (tryptase immunostain counterstained with hematoxylin × 400 magnification) are representative of common findings. The data are presented as columns displaying the means of the total number per high-power field (C) and the relative proportion of degranulated mast cells of all intramucosal mast cells (D) (HPF; magnification × 400) ± standard error (SE). *P < 0.01.

In the immunohistochemical study, esophageal mucosal MCs appeared in the oval or ellipse shapes, distributed mainly in the lamina propria of esophageal mucosa. Cytoplasm was strongly stained brown, while cell nuclei were usually no color. Dropping vacuoles were observed around some MCs (Fig. 1A, B).

### Esophageal mucosal MCs counts

Esophageal mucosal MCs were significantly increased in NERD compared with controls (7.23 ± 2.41 cells/HPF vs 3.79 ± 1.67 cells/HPF, P < 0.01). Moreover, the percentage of degranulated mast cells in NERD was also significantly higher than controls (26.85 ± 8.79% vs 11.50 ± 4.18%, P < 0.01) (Fig. 1C, D).

### Ultrastructure of Esophageal mucosal MCs

**Figure 2:**
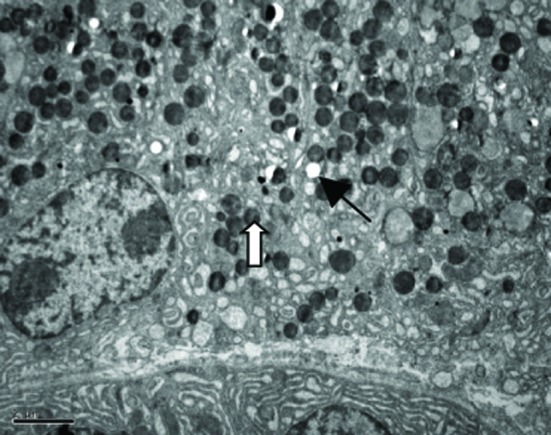
Electron micrograph of a mast cell in the lamina propria of esophageal mucosa in NERD. The cytoplasm of the mast cell showes degranulation with multiple ghost vacuoles (arrows) and targetoid-type granules (open arrows). Original magnification × 6000. Scale bar = 2 µm.

Our TEM study showed clearly MCs with irregular forms lay between the fibrous tissue, more microvilli and folds in the cell surface, more Golgi apparatus, mitochondria and endoplasmic reticulum were found in MCs in patients with NERD, round granules with different sizes and nonuniform densities distributed in cytoplasm. Granules secreting partial mediators had lower density, while granules without mediator secretion had higher density. More vacuoles were left in the cytoplasm after MCs degranulation in patients with NERD. Some secreting granules lay outside the MCs, strongly suggesting MCs activation (Fig. 2).

## Discussion

This study showed that esophageal mucosal MCs were significantly increased in NERD compared with controls (P < 0.01). Moreover, the percentage of degranulated mast cells in NERD was also markedly higher than controls (P < 0.01). These indicate that increased MCs and MCs activation may play roles in the pathogenesis of NERD. Our TEM study demonstrated the increased mucosal MC populations in NERD that showed apparent findings of degranulation, suggesting MCs in esophageal mucosa in NERD were also active, this furthermore confirmed the possible roles of MCs in NERD.

MCs are a potent source of a wide variety of mediators that can mediate diverse proinflammatory, anti-inflammatory and/or immunoregulatory effects. Upon activation, MCs release large quantities of potent biological medicators, such as 5-HT, VIP, NO, and so on, capable of regulating the gastrointestinal motility and visceral perception [5]. 5-HT is generally considered to be the main candidate involved in the modulation of motor and sensory function from the gastrointestinal tract. Administration of Citalopram (a sort of selective serotonin reuptake inhibitors) reduced esophageal sensitivity to distension, but administration of Odansetron (an antagonist of 5-HT_3_ receptor) increased the perceptive thresholds to esophageal balloon distension in the patients with noncardiac chest pain [14]. In addition, the esophageal motility is controlled by excitatory cholinergic “muscarinic” innervations and noncholinergic nonadrenergic inhibitory innervations. The latter is mediated by nitric oxide (NO) and/or vasoactive intestinal polypeptide (VIP). Kassim et al [15] found that serum nitrate and VIP levels were significantly higher in GERD patients than the control group (P < 0.001), and there was a significant positive correlation between the grade of lower end esophagitis and each of serum nitrate and VIP (P < 0.001), as well as between serum nitrate and each of serum VIP, suggesting that abnormal levels of serum VIP and NO may have a role in the pathogenesis of GERD. Exposure of esophageal mucosa to noxious effect of acid refluxed due to the relaxant effect of VIP on lower esophageal sphincter may cause increased NO levels. Moreover, Coelho et al [16] showed that mast cell degranulation initiates a delayed somatic and visceral allodynia, with the participation of serotonin, through 5-HT_1_A receptor activation.

The mechanisms that trigger increased MC numbers and MCs degranulation are not precisely understood. Nakajima et al [17] demonstrated the density of MC is 2 - 3 times greater in Helicobacter pylori (H. pylori) infected than in non-infected subjects. In infected mucosa, mast cells infiltrate between epithelial cells more frequently than in normal mucosa; the intensity of MC infiltration in the mucosa or epithelium significantly correlates with the intensity of neutrophil and mononuclear cell infiltration in this tissue. The increased MC numbers in the gastric mucosa and epithelium in H. pylori-infected gastritis or peptic ulcer patients significantly decrease after H. pylori eradication, they further indicated mast cells undergo degranulation in H. pylori-infected gastric mucosa. In addition, these inflammatory reactions continue until the bacteria are eradicated [18].

Some experimental studies demonstrated that MCs were degranulated with H. pylori-derived products. Kurose et al [19] showed MC degranulation in the vicinity of rat mesenteric venules treated with H. pylori water extract, while Yamamoto et al [20] indicated that H. pylori water extract directly induced degranulation in rat peritoneal MCs. In contrast, Masini et al [21] demonstrated that a cell component of H. pylori enhanced MC degranulation stimulated by compound 48/80, calcium ionophore A23187. Whether mast cells in the human esophageal mucosa degranulate directly under stimulation by H. pylori derived substances remains uncertain.

But the influence of H. pylori infection in the evolution of GERD is controversial since a protective action is identified by some studies [22-24], decreasing the risk or severity of GERD. According to some studies, H. pylori eradication therapy increases acid secretion and promotes the development of GERD and RE. Rebound acid hypersecretion develops after the use of proton pump inhibitors (PPI). Otherwise, nowadays the most widespread opinion is that there is no consistent relationship between GERD and H. pylori infection [25-27].

In addition, Payne et al [28] observed by immnohistochemical staining that MC counts in colonic lamina propria in active ulcerative colonitis was markedly decreased, while eosionphils increased, MCs degranulation may contribute to decreased MC density. However, esophageal mucosal inflammation in NERD is commonly mild and no active lesion, such as swelling of superficial cells, focal basal cell hyperplasia, hyperaemia and dilation of blood vessels in epidermis papilla [29]. These need to be explored in further studies.

In conclusion, alteration of esophageal mucosal MCs was observed and may play important roles in NERD.
